# The Microstructures and Deformation Mechanism of Hetero-Structured Pure Ti under High Strain Rates

**DOI:** 10.3390/ma16217059

**Published:** 2023-11-06

**Authors:** Shuaizhuo Wang, Haotian Yan, Dongmei Zhang, Jiajun Hu, Yusheng Li

**Affiliations:** 1 National Key Laboratory of Transient Physics, Nanjing University of Science and Technology, Nanjing 210094, China; wsz@njust.edu.cn (S.W.); yanhaotian@njust.edu.cn (H.Y.); zbb@njust.edu.cn (D.Z.); hujiajun@njust.edu.cn (J.H.); 2 School of Materials Science and Engineering, Nanjing University of Science and Technology, Nanjing 210094, China

**Keywords:** pure Ti, hetero-structure, deformation mechanism, twinning, high strain rate

## Abstract

This study investigates the microstructures and deformation mechanism of hetero-structured pure Ti under different high strain rates (500 s^−1^, 1000 s^−1^, 2000 s^−1^). It has been observed that, in samples subjected to deformation, the changes in texture are minimal and the rise in temperature is relatively low. Therefore, the influence of these two factors on the deformation mechanism can be disregarded. As the strain rate increases, the dominance of dislocation slip decreases while deformation twinning becomes more prominent. Notably, at a strain rate of 2000 s^−1^, nanoscale twin lamellae are activated within the grain with a size of 500 nm, which is a rarely observed phenomenon in pure Ti. Additionally, martensitic phase transformation has also been identified. In order to establish a correlation between the stress required for twinning and the grain size, a modified Hall–Petch model is proposed, with the obtained value of $K_{twin}$ serving as an effective metric for this relationship. These findings greatly enhance our understanding of the mechanical responses of Ti and broaden the potential applications of Ti in dynamic deformation scenarios.

## 1. Introduction

The usage of pure Ti has been steadily increasing in the fields of chemical, biomedical, and aerospace engineering. This is primarily due to its exceptional corrosion resistance, excellent biological compatibility, and impressive high specific strength [1,2]. As a structural material, pure Ti not only encounters static loads, but also confronts challenges from high-speed impact loads. Structural components in industries such as aerospace and defense industries may inevitably be involved in high-speed collision events [3]. Compared to static loading, the mechanical properties and deformation mechanisms of titanium and its alloys undergo significant changes under dynamic loading conditions, especially at high strain rates [4]. Therefore, conducting a thorough investigation of the deformation mechanism of pure Ti at high strain rates is of great significance for promoting the extensive utilization of pure Ti.

Previous studies have indicated that the deformation mechanism of pure Ti are affected by various factors, including its inherent characteristics (grain size, alloying elements, texture, etc.) and experimental conditions (loading mode, strain rate, temperature, strain level, etc.) [5,6,7,8,9]. In particular, strain rate plays a significant role and greatly influences the deformation behavior of materials. It has been found that higher strain rate facilitates the activation of twinning [10,11]. This can be attributed to the fact that a high strain rate promotes the rapid accumulation of dislocations in localized regions, leading to severe stress concentration. These regions of stress concentration provide effective nucleation sites for twinning [12]. It is worth noting that at high strain rates, the heat dissipation process in titanium is relatively slower compared to heat generation. This results in thermal softening during the accumulation of plastic deformation [4]. Consequently, instabilities in plastic flow and the formation of shear bands are observed, exerting a significant impact on the deformation mechanism. In addition, the influence of crystallographic texture on the mechanical behavior and deformation mechanism of pure Ti cannot be underestimated, owing to its hexagonal close-packed (HCP) structure. Extensive research has revealed that the presence of two distinctly different initial textures results in varying mechanical responses [13].

In addition to strain rate, the deformation mechanism of pure Ti also has a strong grain size dependency [14]. Studies have revealed that the occurrence of twinning becomes increasingly challenging as the grain size decreases, reaching a critical threshold at 750 nm. Below this grain size, deformation twinning is completely replaced by dislocation slip. Nevertheless, it is important to note that this conclusion solely takes into account the influence of static loads [15]. Currently, high-speed deformation studies of pure Ti primarily focus on samples with large grain sizes, where deformation are mainly dominated by dislocation slips and various types of twinning [13,16]. Unfortunately, there has been limited experimental research conducted on the high-speed deformation of pure Ti with hierarchical grain size, thereby impeding an in-depth investigation into grain size effect. The reason for this predicament is the difficulty in fabricating cross-scale structures with varying grain sizes. In recent years, an innovative approach, microstructural heterogenization, offering a promising avenue to overcome this challenge. This strategic paradigm has demonstrated a remarkable synergy in enhancing mechanical properties in pure Ti [17]. By incorporating heterogenization in grain size, ranging from coarse to ultra-fine regime, this structure can be served as an ideal model for studying the mechanism change during high-speed deformation processes.

Therefore, this study focuses on hetero-structured pure Ti samples with a hierarchical grain size. A room temperature split Hopkinson pressure bar test was conducted to examine the dynamic mechanical responses of the samples at different strain rates. Microstructural evolution during deformation was characterized using electron backscattering diffraction (EBSD) and transmission electron microscopy (TEM) techniques. Microstructures and deformation mechanism of hetero-structured pure Ti were systematically investigated, with a particular focus on exploring the influence of grain size on twinning under high strain rates.

## 2. Experiment

**Figure 1:**
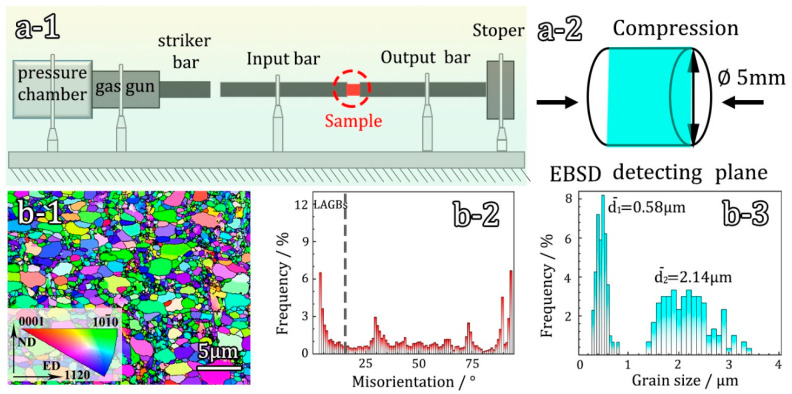
(**a-1**) The schematics of the split Hopkinson pressure bar system; (**a-2**) specimen; (**b-1**–**b-3**) EBSD characterization of the initial hetero-structured sample. (**b-1**) Inverse polar figure (IPF) map; (**b-2**) distribution of grain boundary orientation; (**b-3**) distributions of grain size.

**Figure 2:**
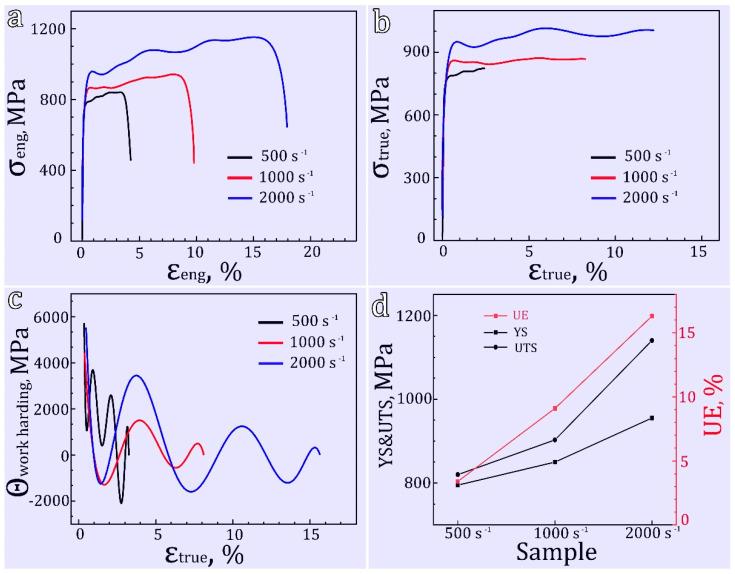
Mechanical properties of hetero-structured samples under different strain rate: (**a**) compressive engineering stress-strain curves; (**b**) true stress-strain curves from compressive tests; (**c**) strain hardening rate curves; (**d**) corresponding yield strength (YS), ultimate compressive strength (UCS) and compression strain of the samples under different strain rate.

**Figure 3:**
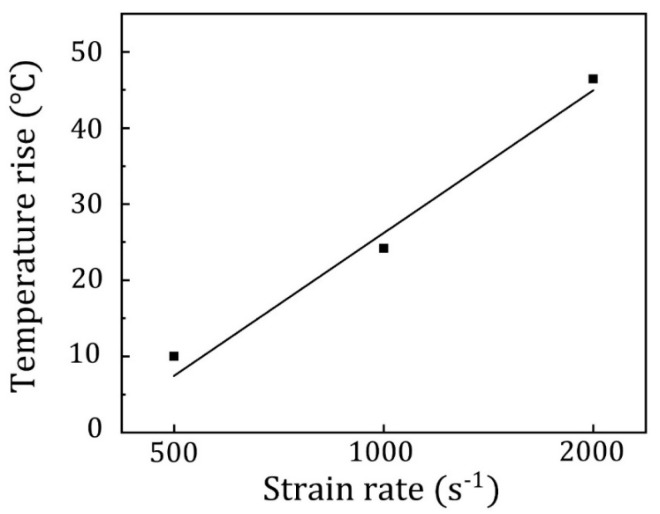
Adiabatic temperature rises values for overall hetero-structured samples under different strain rate.

In this study, a split Hopkinson pressure bar (ALT1000) compression test was conducted to analyze the mechanical responses of the sample. The compression was performed along the ND (normal direction) axis of Ti samples at room temperature, and the compression rate was controlled by adjusting the gas pressure. This allowed for data collection on the dynamic mechanical responses of the samples at different strain rates (500 s^−1^, 1000 s^−1^, 2000 s^−1^). Figure 1(a-1) illustrates a schematic diagram of the Hopkinson impact test, while Figure 1(a-2) displays the observation surface of the sample in the transverse direction (TD) plane. Commercially pure Ti (grade 1) was selected as the material for this investigation, and the compositions is as follows (wt%): 0.001 C, 0.005 N, 0.0015 H, 0.085 O, 0.045 Fe, and balanced Ti. To prepare the initial hetero-structured pure Ti samples with hierarchical grain sizes, a combination of rolling and annealing techniques was employed. The Ti samples underwent cold rolling to an 80% reduction in thickness, followed by annealing at 480 °C for 5 min under vacuum conditions. EBSD observations were carried out using a Zeiss Auriga scanning electron microscope (SEM) with an acceleration voltage of 15 kV and a step size of 50 nm. TEM characterization was performed using an FEI TEM at 200 kV. Figure 1(b-1) presents the EBSD results of the hetero-structured pure Ti sample. The grain size exhibits a bimodal distribution, with an average grain size of 580 nm for the ultrafine grains and 2.14 μm for the coarse grains (Figure 1(b-3)). No twinning was observed in the sample. The grain boundary orientation distribution map (Figure 1(b-2)) reveals that the proportion of low angle grain boundaries (LAGBs) in the sample is approximately 19.5%. This indicates that the material has undergone significant dislocation recovery during the annealing process.

## 3. Results and Discussion

Figure 2a presents the Hopkinson impact mechanical curves of the hetero-structured pure Ti samples deformed under strain rates of 500 s^−1^, 1000 s^−1^, 2000 s^−1^. It can be observed that the dynamic compressive stress gradually increases as the strain increases, indicating that the sample absorbs impact energy during the impact process. It is important to note that the rapid decline in flow stress at the end of the curve is solely attributed to the termination of the applied load on the Hopkinson bar and not due to material failure. To obtain the true stress-strain curve, it can be calculated based on the compressive engineering stress-strain curve, as depicted in Figure 2b. This relationship can be described as follows:

(1) $$\sigma_{t}=\sigma_{e}\left(1-\varepsilon_{e}\right)$$

(2) $$\varepsilon_{t}=-In\left(1-\varepsilon_{e}\right)$$

where $\sigma_{t}$ and $\varepsilon_{t}$ represent the true stress and strain, respectively, $\sigma_{e}$ and $\varepsilon_{e}$ represent engineering stress and strain, respectively. It can be observed that the increase in true stress is not significant with increasing strain, but there is a noticeable oscillatory pattern. This oscillatory pattern arises from stress vibrations, which occur when an elastic wave, caused by an impact from a hammer in the test machine, propagates through the test sample and is detected by the load cell [18]. Figure 2c shows the strain hardening capacity of hetero-structured sample under different strain rates, and this is closely related to the deformation mechanism. In Figure 2d, the variation trends of different mechanical performance indicators with strain rate are illustrated. It can be seen that the corresponding yield strength (YS), ultimate compressive strength (UCS), and uniform elongation (UE) increase with the increase in strain rate.

During high-speed deformation, a substantial amount of the energy involved in plastic deformation is converted into heat energy. Due to insufficient heat dissipation, a noticeable local temperature rise occurs, leading to thermal softening of the material. The influence of temperature on the deformation mechanism of the material is crucial, thus necessitating a thorough examination of the impact of temperature rise during high-speed deformation. If we assume that all the energy generated from plastic deformation is completely transformed into heat energy and not dissipated, the resulting adiabatic temperature rise can be expressed as:

(3) $$\Delta T=\frac{\beta }{\rho C_{v}}\int_{0}^{\varepsilon_{f}}\sigma_{T}d\varepsilon_{T}$$

where ρ represents density and $C_{v}$ refers to specific heat capacity, $\sigma_{t}$ and $\varepsilon_{t}$ represent the true stress and strain, respectively, and $\varepsilon_{f}\;$ is the max strain. β is a constant known as the Taylor–Quinney factor, which denotes the coefficient of plastic deformation work converted into heat. The values of ρ, $C_{v}$, β are ρ= 4.51 g/cm^3^, $C_{v}=$ 527 J/(kg·°C), β = 0.9 [19]. The results calculated from Equation (3) are shown in Figure 3. At a strain rate of 500 s^−1^, the theoretical temperature rise is approximately 10 K. As the impact rate increases, the total strain also increases, resulting in an increase in the plastic deformation energy of the corresponding sample and a subsequent increase in adiabatic temperature rise. When the strain rate reaches 2000 s^−1^, the corresponding theoretical temperature rise is approximately 45 K. Although the temperature rise increases obviously with the strain rate, it is still far from reaching the recrystallization temperature. Therefore, we believe that the temperature rise here has little effect on the deformation mechanism, and the emphasis should be placed on the effect of stress.

**Figure 4:**
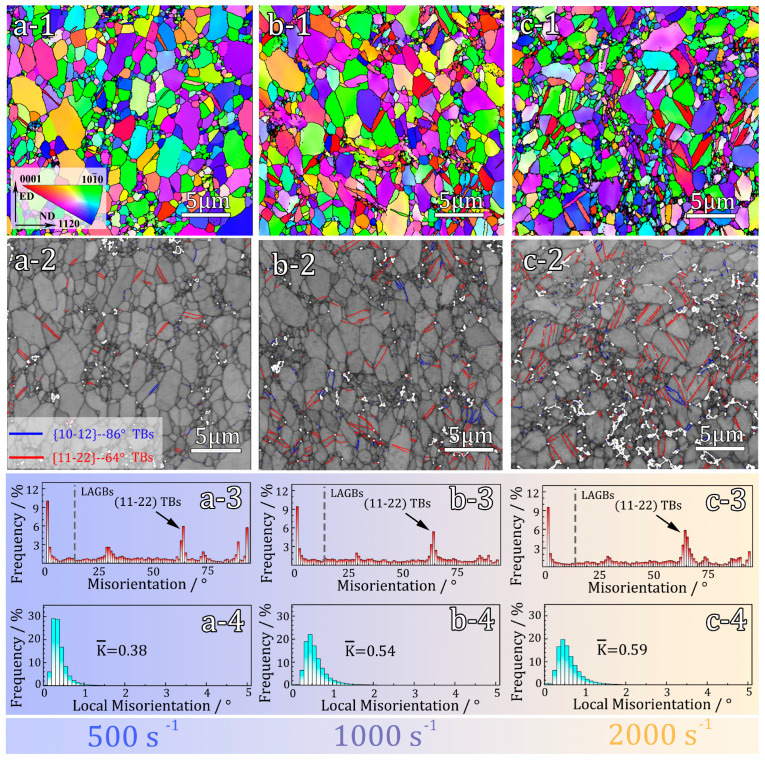
EBSD observations of deformed hetero-structured sample under a strain rate of 500 s^−1^, 1000 s^−1^, 2000 s^−1^, respectively: (**a-1**–**c-1**) IPF map; (**a-2**–**c-2**) twin boundary map; (**a-3**–**c-3**) distribution of grain boundary orientation; (**a-4**–**c-4**) statistical distributions of Local Misorientation.

Figure 4 presents the EBSD results of hetero-structured pure Ti subjected to various strain rates. From the IPF maps depicted in Figure 4(a-1–c-1), it is evident that the microstructure remains its hetero-structured nature, with no signs of recrystallization or significant grain refinement within the samples. However, after the impact tests, a noticeable occurrence of twinning is observed. Through analyzing the orientation differences (Figure 4(a-3–c-3), it can be observed that the predominant twinning type in the three deformed samples is {$11\bar{2}2$} compression twins [20]. For pure Ti with an HCP structure, {$11\bar{2}2$} twinning is more readily activated when the lattice experiences stress along the c-axis [21]. As depicted in the twin boundary map (Figure 4(a-2–c-2)), an increase in the percentage of twin boundaries, from 5.4% to 6.2% and 8.6%, is observed when the strain rate increases from 500 s^−1^ to 1000 s^−1^ and 2000 s^−1^. The percentage of twin boundaries is determined by calculating the areal fractions of twin boundaries among all interfaces within the EBSD maps. It is noteworthy that at relatively low strain rates (500 s^−1^), twinning only occurs in coarse grains due to the notable influence of grain size on twinning behavior in pure Ti [22,23]. When the grain size is refined to the ultrafine range, twinning is nearly absent. However, when the strain rate is increased to 2000 s^−1^, twinning appears in some smaller grains, as shown in Figure 4(c-2). This occurrence could be attributed to the unique microstructure of hetero-structured pure Ti and the high stress induced by the ultra-high strain rate impact, which triggers twinning [24]. Additionally, the proportion of LAGBs does not vary significantly under different strain rates, implying a decreased dominance of dislocations in the impact deformation process [25]. Figure 4(a-4–c-4) illustrate the statistical distribution plots of local misorientation corresponding to impact deformation at different strain rates. By analyzing these plots, the average Local Misorientation difference ($\bar{K}$) can be calculated. For strain rates of 500 s^−1^, 1000 s^−1^, and 2000 s^−1^, the $\bar{K}$ values are 0.38, 0.54, and 0.59, respectively. An increase in $\bar{K}$ indicates a higher level of plastic deformation or a higher density of defects in the sample [26]. Specifically, the sample subjected to a strain rate of 2000 s^−1^ shows a similar $\bar{K}$ to the sample at a strain rate of 1000 s^−1^, suggesting insignificant dislocation accumulation as the strain rate increases from 1000 s^−1^ to 2000 s^−1^. This observation is consistent with the minimal increase in the proportion of LAGBs shown in Figure 4(b-2,c-2).

**Figure 5:**
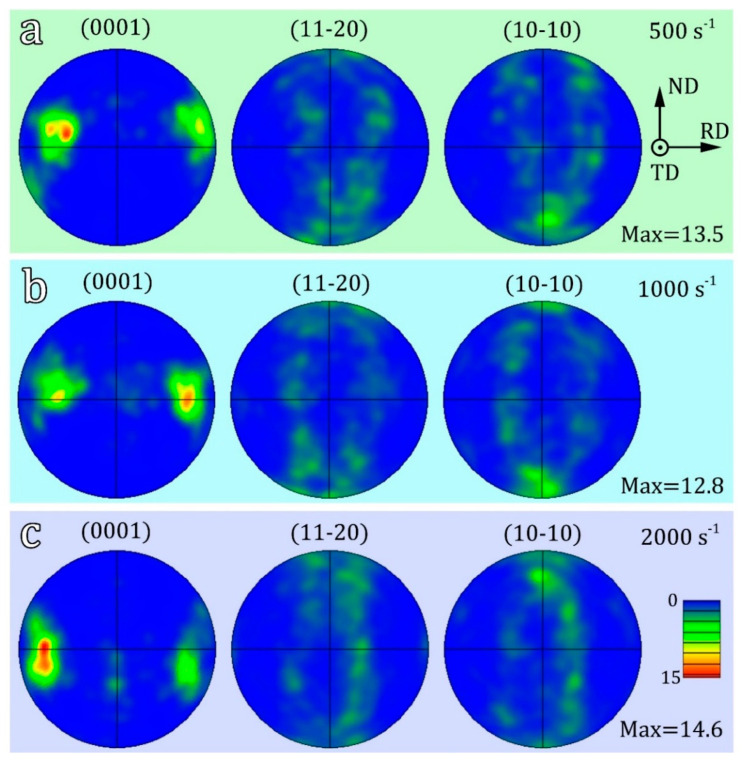
(0001), (11$\bar{2}$0) and (10$\bar{1}$0) pole figures of deformed hetero-structured samples under different strain rate: (**a**) 500 s^−1^, (**b**) 1000s^−1^, (**c**) 2000 s^−1^.

Figure 5a–c display the pole figures that correspond to the samples deformed under strain rates of 500 s^−1^, 1000 s^−1^, and 2000 s^−1^, respectively. The pole figures reveal a bimodal basal texture, with the highest texture strengths measured at 13.5, 12.8, and 14.6, respectively. There is no significant change in the texture type when compared to the original sample. These results indicate that the strain rate has a minimal effect on the texture. In other words, the influence of texture on the change in deformation mechanism can be ignored.

**Figure 6:**
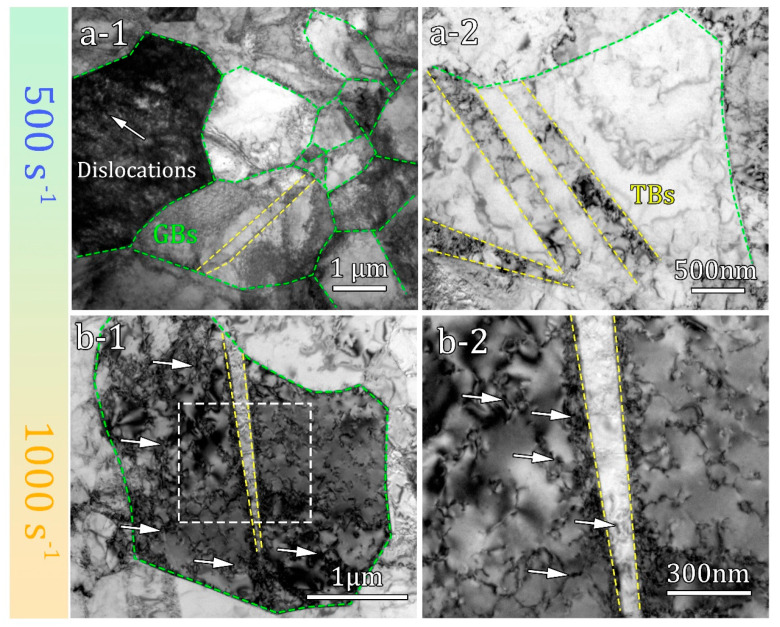
TEM images of deformed hetero-structured sample with different strain rates. (**a-1**,**a-2**) 500 s^−1^; (**b-1**,**b-2**) 1000 s^−1^, The white arrows indicate dislocations.

Figure 6(a-1,a-2) shows a bright-field TEM image of Ti sample deformed at a strain rate of 500 s^−1^. It can be observed that the deformed sample contains a high density of dislocations, which is a typical characteristic of plastic deformation. Twin boundaries are commonly observed within coarse grains, as illustrated by the yellow dotted lines in Figure 6(a-1,a-2). In contrast, no twinning is observed in the ultrafine grains, where dislocation slip serves as the primary mode of deformation [27]. When the impact strain rate increases to 1000 s^−1^, the microstructure of the sample is shown in Figure 6(b-1,b-2), revealing a typical deformed structure characterized by a significant presence of defects such as dislocations and twins. Similarly, no twinning is observed in the ultrafine grain region of the sample. This implies that even at a strain rate of 1000 s^−1^, twinning remains suppressed in the ultrafine grains. On the other hand, within the coarse grains, a prominent twin boundary is present, accompanied by a significant distribution of dislocations that form a ring-like pattern. In the magnified region (Figure 6(b-2)), a large number of dislocations can be seen near the twin boundary, indicating that the newly formed twin boundary inhibits the movement of dislocations [28]. Additionally, dislocations are also observed within the twin lamellae.

**Figure 7:**
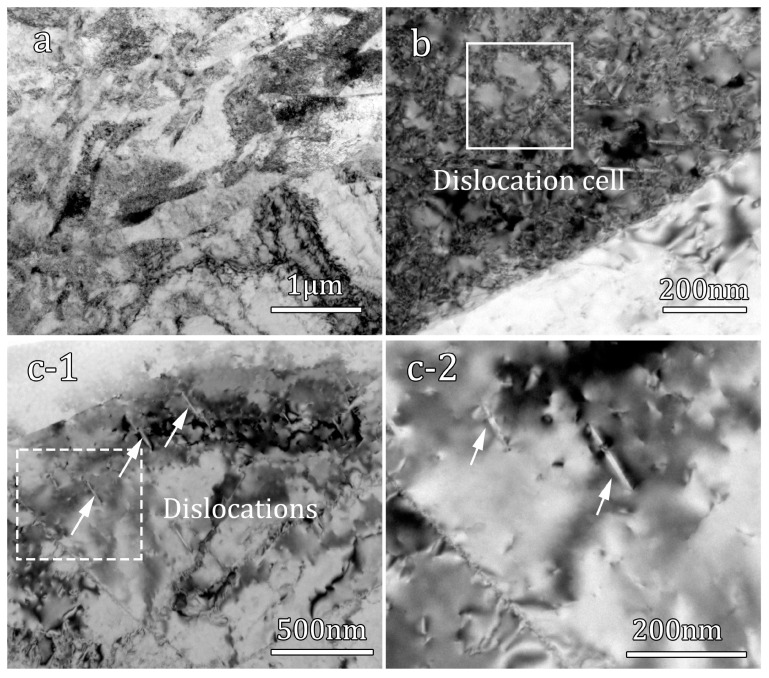
Microstructure of deformed hetero-structured sample with a strain rate of 2000 s^−1^: (**a**,**b**) bright field TEM images; (**c-1**) The TEM image of martensitic phases; (**c-2**) close-up view of the area marked by the white dash line box in (**c-1**), the white arrows indicate needle-like martensitic phases.

When the impact strain rate reaches 2000 s^−1^; the microstructure undergoes severe plastic deformation, leading to significant disordering within the grains, as shown in Figure 7. Consequently, the grains exhibit a remarkably high density of defects, making it difficult to clearly distinguish grain boundaries and twin boundaries based solely on morphology (Figure 7a,b). Although it has been verified by EBSD observation (Figure 4(c-2)) that the areal twin percentage is 8.6%, TEM bright-field images at high magnification reveal the formation of dislocation cell structures in certain regions, as depicted by the solid rectangle in Figure 7b. Additionally, Figure 7(c-1,c-2) show the presence of numerous fine needle-like structures, which were not found in the deformed samples at strain rates of 500 s^−1^ and 1000 s^−1^. These structures resemble the needle-like martensitic phases found in titanium alloys. Previous studies have indicated that under extreme deformation conditions, pure Ti can undergo stress-induced martensitic phase transformation, transitioning from the HCP phase to the face-centered cubic (FCC) phase, ultimately resulting in the formation of fine needle-like martensitic structures [29,30].

For the hetero-structured pure Ti investigated in this study, the grain sizes are generally below 3 μm and contain a significant number of ultrafine grains, resulting in a relatively small grain size (Figure 1(b-3)). Considering the influence of grain size on deformation mechanism, dislocation slip is the most prevalent deformation carrier under this grain size [31,32]. On one hand, higher strain rates lead to the activation of more dislocation sources, resulting in the entanglement of dislocations and impeding their motion. This leads to the accumulation and pile-up of a significant number of dislocations, as confirmed by TEM images of the deformed samples. On the other hand, the hierarchical structure contains numerous hetero-structure interfaces. These interfaces give rise to hetero-deformation-induced (HDI) stress, which in turn leads to a significant accumulation of geometrically necessary dislocations [33,34].

**Figure 8:**
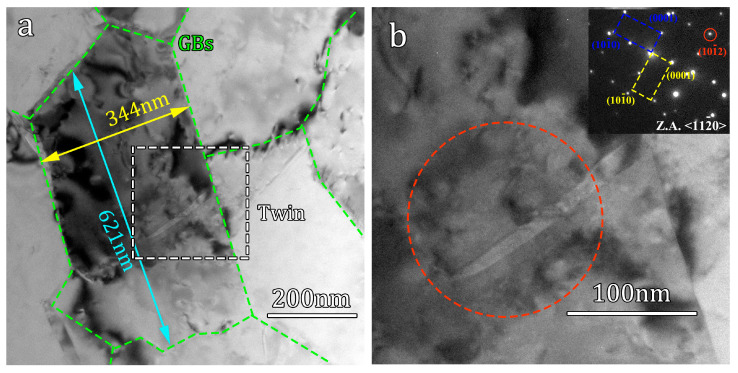
TEM images of ultra-fine grain of deformed hetero-structured sample under a strain rate of 2000 s^−1^: (**a**) bright field TEM image of ultra-fine grain; (**b**) close-up view of the area marked by the white dash line box in (**a**), and the corresponding selected area diffraction pattern.

It is generally believed that twinning is unlikely to occur in ultrafine grains [22,31]. However, in the deformed samples under a strain rate of 2000 s^−1^, TEM bright-field images reveal the presence of small nanoscale twin lamellae within the ultrafine grains with an equivalent size of approximately 500 nm, as shown in Figure 8. The thickness of these twin lamellae is approximately 10 nm. The occurrence of twinning within the ultrafine grains is not widespread, but rather localized, observed in specific individual grains. This localized twinning may be due to the inhomogeneous deformation during impact, which leads to highly localized stress levels that satisfy the nucleation conditions for twinning within the ultrafine grains [35,36]. The significance of this discovery lies in gaining a profound understanding of the fundamental reasons behind the inhibitory effect of grain refinement on twinning. It uncovers that twinning is not completely absent within ultrafine grains, but rather occurs under extremely stringent conditions.

Numerous studies have demonstrated that dislocations play a pivotal role in the initiation of twinning in pure Ti [37,38,39]. Furthermore, the Hall–Petch relationship, which is based on the principle of dislocation obstacle at grain boundaries, has effectively explained the origin of strength [15]. Hence, we hypothesize that the correlation between the required stress for twin activation and the grain size is similar to the Hall–Petch relationship. Consequently, in order to gain a better understanding of the emergence of nanoscale twin lamellae in ultrafine grain, we propose a modified Hall–Petch relationship, which takes into account the fact that the stress required to initiate deformation twinning, $\sigma_{twin},$ is inversely proportional to the square root of grain size. This the relationship can be described as:

(4) $$\sigma_{twin}=K_{twin}\times d^{-\frac{1}{2}}$$

where d represent the average grain size and $K_{twin}$ is a constant that represents the sensitivity of twin activation to grain size. A higher $K_{twin}$ value indicates a stronger grain size effect on twinning. The experimental results in this study demonstrate that twinning is activated at an equivalent grain size of 500 nm (the finest observable grain size where twinning occurs) when the strain rate reached 2000 s^−1^. Based on the stress–strain curve (Figure 2), the corresponding range of flow stress is between 0.92 GPa and 1.04 GPa. According to Equation (4), the $K_{twin}$ can be calculated to range from 0.651 MNm^−3/2^ to 0.735 MNm^−3/2^, which is significantly larger than the *K* value obtained from the traditional Hall–Petch relationship (0.24 MNm^−3/2^) [40]. Extensive research has indicated that the critical shear stress for compression twinning in pure Ti falls within the range of 125 MPa to 255 MPa when the grain size is between 10 μm and 50 μm [41,42,43]. To validate the equation, by assuming values of $K_{twin}$ as 0.651 and 0.735, when $K_{twin}$ is set to 0.651, the critical shear stress for twinning at grain sizes ranging from 10 μm to 50 μm can be calculated as 92 MPa to 203 MPa. On the other hand, when $K_{twin}$ is set to 0.735, the critical shear stress can be calculated as 105 MPa to 232 MPa. These values are in good agreement with previous studies, confirming the scientific validity of this equation. The value of $K_{twin}\;$ reported in this study can be used to predict the stress required to initiate deformation twinning across a wide range of grain sizes spanning from coarse to ultrafine.

In summary, our experimental results and empirical model greatly enhance our understanding of the twinning mechanism at ultrafine grain level, as well as establish a correlation between grain size and critical shear stress. These findings also offer potential avenues for strengthening mechanisms through twinning in HCP structured materials with ultrafine grains.

## 4. Conclusions

This study investigates the influence of deformation rate on the microstructures and deformation mechanism in hetero-structured pure Ti. The results can be summarized as follows:

1. The mechanical responses and deformation mechanism of hetero-structured pure Ti samples are closely related to the strain rate. As the strain rate increases from 500 s^−1^ to 2000 s^−1^, dislocation activities are the primary deformation carrier, but the dominance of dislocation slipping reduces and the dislocation configurations undergo changes. Conversely, there is an increase in the percentage of deformation twinning with higher strain rates.
2. It has been found that under different high deformation rates (500 s^−1^, 1000 s^−1^, 2000 s^−1^), the changes in texture are relatively minimal, and the degree of temperature rise is low (far lower than the recrystallization temperature). Consequently, the alterations in these two influential factors are insufficient to induce changes in the deformation mechanism.
3. When subjected to a strain rate of 2000 s^−1^, martensitic phase transformation is identified in the deformed Ti sample. Moreover, nanoscale twin lamellae are observed within the ultrafine grain, which can be attributed to the high flow stress. A modified Hall–Petch model is proposed, and the obtained $K_{twin}$ can be used to effectively establish the correlation between the stress required for twinning and the grain size.

## Data Availability

The data that support the findings of this study are available from the corresponding author, upon reasonable request.

## References

[1] Özcan M., Hämmerle C. (2012). Titanium as a Reconstruction and Implant Material in Dentistry: Advantages and Pitfalls. Materials.

[2] Konstantinov A.S., Bazhin P.M., Stolin A.M., Kostitsyna E.V., Ignatov A.S. (2018). Ti-B-Based Composite Materials: Properties, Basic Fabrication Methods, and Fields of Application (Review). Compos. Part A Appl. Sci. Manuf..

[3] Zochowski P., Bajkowski M., Grygoruk R., Magier M., Burian W., Pyka D., Bocian M., Jamroziak K. (2021). Ballistic Impact Resistance of Bulletproof Vest Inserts Containing Printed Titanium Structures. Metals.

[4] Yan N., Li Z., Xu Y., Meyers M.A. (2021). Shear Localization in Metallic Materials at High Strain Rates. Prog. Mater. Sci..

[5] Lei L., Zhao Q., Zhao Y., Wu C., Huang S., Jia W., Zeng W. (2022). Gradient nanostructure, phase transformation, amorphization and enhanced strength-plasticity synergy of pure titanium manufactured by ultrasonic surface rolling. J. Am. Acad. Dermatol..

[6] Wang M., Wang Y., He Q., Wei W., Guo F., Su W., Huang C. (2022). A Strong and Ductile Pure Titanium. Mater. Sci. Eng. A.

[7] Won J.W., Lee S., Kim W.C., Hyun Y.-T., Lee D.W. (2023). Significantly increased twinning activity of pure titanium during room-temperature tensile deformation by cryogenic-deformation treatment. Mater. Sci. Eng. A.

[8] Zhao S., Zhang R., Yu Q., Ell J., Ritchie R.O., Minor A.M. (2021). Cryoforged Nanotwinned Titanium with Ultrahigh Strength and Ductility. Science.

[9] Huang Z., Cao Y., Nie J., Zhou H., Li Y. (2018). Microstructures and Mechanical Properties of Commercially Pure Ti Processed by Rotationally Accelerated Shot Peening. Materials.

[10] Zhou P., Xiao D., Jiang C., Sang G., Zou D. (2016). Twin Interactions in Pure Ti Under High Strain Rate Compression. Met. Mater. Trans. A.

[11] Jia H., Marthinsen K., Li Y. (2019). Revealing Abnormal {112¯1} Twins in Commercial Purity Ti Subjected to Split Hopkinson Pressure Bar. J. Alloys Compd..

[12] Wang T.B., Li B.L., Li M., Li Y.C., Nie Z.R. (2014). The Dynamic Mechanical Behavior and Microstructural Evolution of Commercial Pure Titanium. Adv. Mater. Res..

[13] Gurao N., Kapoor R., Suwas S. (2011). Deformation behaviour of commercially pure titanium at extreme strain rates. Acta Mater..

[14] Deguchi M., Yamasaki S., Mitsuhara M., Nakashima H., Tsukamoto G., Kunieda T. (2023). Tensile Deformation Behaviors of Pure Ti with Different Grain Sizes under Wide-Range of Strain Rate. Materials.

[15] Ovid’Ko I., Valiev R., Zhu Y. (2018). Review on superior strength and enhanced ductility of metallic nanomaterials. Prog. Mater. Sci..

[16] Xu F., Zhang X., Ni H., Liu Q. (2012). Deformation twinning in pure Ti during dynamic plastic deformation. Mater. Sci. Eng. A.

[17] Wu X., Yang M., Yuan F., Wu G., Wei Y., Huang X., Zhu Y. (2015). Heterogeneous lamella structure unites ultrafine-grain strength with coarse-grain ductility. Proc. Natl. Acad. Sci. USA.

[18] Tanaka Y., Kondo M., Miyazaki N., Ueji R. (2010). Deformation Behavior of Pure Titanium at a Wide Range of Strain Rates. J. Phys. Conf. Ser..

[19] Li Q., Xu Y.B., Bassim M.N. (2004). Dynamic Mechanical Behavior of Pure Titanium. J. Mater. Process. Technol..

[20] Xu S., Wang J. (2022). Deformation Twins Stimulated by {$11\bar{2}2$} Twinning in Adjacent Grain in Titanium. Acta Mater..

[21] Huang Z., Jin S., Zhou H., Li Y., Cao Y., Zhu Y. (2019). Evolution of twinning systems and variants during sequential twinning in cryo-rolled titanium. Int. J. Plast..

[22] Yu Q., Mishra R.K., Minor A.M. (2012). The Effect of Size on the Deformation Twinning Behavior in Hexagonal Close-Packed Ti and Mg. JOM.

[23] Li L., Zhang Z., Shen G. (2015). Effect of Grain Size on the Tensile Deformation Mechanisms of Commercial Pure Titanium as Revealed by Acoustic Emission. J. Mater. Eng. Perform..

[24] Yu K., Wang X., Mahajan S., Beyerlein I.J., Cao P., Rupert T.J., Schoenung J.M., Lavernia E.J. (2023). Twin Nucleation from Disconnection-Dense Sites between Stacking Fault Pairs in a Random Defect Network. Materialia.

[25] Mittemeijer E.J. (2021). The Crystal Imperfection; Structure Defects. Fundamentals of Materials Science.

[26] Wei K., Hu R., Yin D., Xiao L., Pang S., Cao Y., Zhou H., Zhao Y., Zhu Y. (2021). Grain size effect on tensile properties and slip systems of pure magnesium. Acta Mater..

[27] Huang Z., Wen D., Hou X., Li Y., Wang B., Wang A. (2022). Grain size and temperature mediated twinning ability and strength-ductility correlation in pure titanium. Mater. Sci. Eng. A.

[28] Ahmadikia B., Wang L., Kumar M.A., Beyerlein I.J. (2023). Grain boundary slip—Twin transmission in titanium. Acta Mater..

[29] Zhao H., Ding N., Ren Y., Xie H., Yang B., Qin G. (2019). Shear-induced hexagonal close-packed to face-centered cubic phase transition in pure titanium processed by equal channel angular drawing. J. Mater. Sci..

[30] Zheng X., Gong M., Xiong T., Ge H., Yang L., Zhou Y., Zheng S., Wang J., Ma X. (2019). Deformation Induced Fcc Lamellae and Their Interaction in Commercial Pure Ti. Scr. Mater..

[31] Sun J., Trimby P., Yan F., Liao X., Tao N., Wang J. (2013). Grain size effect on deformation twinning propensity in ultrafine-grained hexagonal close-packed titanium. Scr. Mater..

[32] Palán J., Procházka R., Džugan J., Nacházel J., Duchek M., Németh G., Máthis K., Minárik P., Horváth K. (2018). Comprehensive Evaluation of the Properties of Ultrafine to Nanocrystalline Grade 2 Titanium Wires. Materials.

[33] Zhu Y., Wu X. (2023). Heterostructured Materials. Prog. Mater. Sci..

[34] Zhu Y., Ameyama K., Anderson P.M., Beyerlein I.J., Gao H., Kim H.S., Lavernia E., Mathaudhu S., Mughrabi H., Ritchie R.O. (2020). Heterostructured materials: Superior properties from hetero-zone interaction. Mater. Res. Lett..

[35] Xu S., Zhou P., Liu G., Xiao D., Gong M., Wang J. (2019). Shock-Induced Two Types of {$10\bar{1}2$} Sequential Twinning in Titanium. Acta Mater..

[36] He Y., Li B., Wang C., Mao S.X. (2020). Direct observation of dual-step twinning nucleation in hexagonal close-packed crystals. Nat. Commun..

[37] Kou Z., Yang Y., Huang B., Luo X., Li P., Zhao G., Zhang W. (2017). Observing the Dynamic {$10\bar{1}1$} twining Process in Pure Ti at Atomic Resolution. Scr. Mater..

[38] Liao X., Wang J., Nie J., Jiang Y., Wu P. (2016). Deformation twinning in hexagonal materials. MRS Bull..

[39] Paudel Y., Giri D., Priddy M.W., Barrett C.D., Inal K., Tschopp M.A., Rhee H., El Kadiri H. (2021). A Review on Capturing Twin Nucleation in Crystal Plasticity for Hexagonal Metals. Metals.

[40] Zherebtsov S., Dyakonov G., Salem A., Sokolenko V., Salishchev G., Semiatin S. (2013). Formation of nanostructures in commercial-purity titanium via cryorolling. Acta Mater..

[41] Wang L., Zheng Z., Phukan H., Kenesei P., Park J.-S., Lind J., Suter R., Bieler T. (2017). Direct measurement of critical resolved shear stress of prismatic and basal slip in polycrystalline Ti using high energy X-ray diffraction microscopy. Acta Mater..

[42] Cao Y., Ni S., Liao X., Song M., Zhu Y. (2018). Structural evolutions of metallic materials processed by severe plastic deformation. Mater. Sci. Eng. R Rep..

[43] Yang H., Li H., Ma J., Wei D., Chen J., Fu M. (2020). Temperature dependent evolution of anisotropy and asymmetry of α-Ti in thermomechanical working: Characterization and modeling. Int. J. Plast..

